# Construction of an infectious cloning system of porcine reproductive and respiratory syndrome virus and identification of glycoprotein 5 as a potential determinant of virulence and pathogenicity

**DOI:** 10.3389/fmicb.2023.1227485

**Published:** 2023-07-20

**Authors:** Yuqing Wei, Guo Dai, Mei Huang, Lianghai Wen, Rui Ai Chen, Ding Xiang Liu

**Affiliations:** ^1^Guangdong Province Key Laboratory Microbial Signals and Disease Control, Integrative Microbiology Research Centre, South China Agricultural University, Guangzhou, Guangdong, China; ^2^Zhaoqing Branch Center of Guangdong Laboratory for Lingnan Modern Agricultural Science and Technology, Zhaoqing, Guangdong, China; ^3^Zhaoqing Institute of Biotechnology Co., Ltd., Zhaoqing, Guangdong, China

**Keywords:** porcine reproductive and respiratory syndrome virus, infectious clone, GP5, cytokines, pathogenicity and virulence

## Abstract

Porcine reproductive and respiratory syndrome virus (PRRSV) infection of pigs causes a variety of clinical manifestations, depending on the pathogenicity and virulence of the specific strain. Identification and characterization of potential determinant(s) for the pathogenicity and virulence of these strains would be an essential step to precisely design and develop effective anti-PRRSV intervention. In this study, we report the construction of an infectious clone system based on PRRSV vaccine strain SP by homologous recombination technique, and the rescue of a chimeric rSP-HUB2 strain by replacing the GP5 and M protein-coding region from SP strain with the corresponding region from a highly pathogenic strain PRRSV-HUB2. The two recombinant viruses were shown to be genetically stable and share similar growth kinetics, with rSP-HUB2 exhibiting apparent growth and fitness advantages. Compared to in cells infected with PRRSV-rSP, infection of cells with rSP-HUB2 showed significantly more inhibition of the induction of type I interferon (IFN-β) and interferon stimulator gene 56 (ISG56), and significantly more promotion of the induction of proinflammatory cytokines IL-6, IL-8, ISG15 and ISG20. Further overexpression, deletion and mutagenesis studies demonstrated that amino acid residue F16 in the N-terminal region of the GP5 protein from HUB2 was a determinant for the phenotypic difference between the two recombinant viruses. This study provides evidence that GP5 may function as a potential determinant for the pathogenicity and virulence of highly pathogenic PRRSV.

## Introduction

Porcine reproductive and respiratory syndrome (PRRS) is a highly infectious disease of pigs characterized as ‘blue-ear’ pig disease (Terpstra et al., 1991; Shabir et al., 2016). The etiologic agent, porcine reproductive and respiratory syndrome virus (PRRSV), is an enveloped and positive-stranded RNA virus (Snijder et al., 2013). Its genomic RNA (gRNA) is approximately 15 kb in length and encodes 11 known open reading frames (ORFs). Two large ORFs, 1a and 1b, code for the nonstructural replicase proteins (nsps) that play essential roles in the viral replication cycle, including rearrangement of host membranes to establish viral replication complexes (RC), replication of gRNA and transcription of subgenomic RNAs (sgRNAs) for the efficient expression of viral proteins (Pasternak et al., 2001; Yuan et al., 2004). ORF2a, ORF2b, ORF3-7 and the recently discovered ORF5a encode structural proteins GP2, E, GP3, GP4, GP5, matrix protein (M), nucleocapsid protein (N) and GP5a, respectively, constituting the structural protein components of PRRSV virions. As small envelope proteins and secondary structural proteins, GP2, GP3 and GP4 interact to form heterotrimers, and together with E proteins, assist viral infectivity, bind to cell receptors, and effectively induce neutralizing antibody production and cellular immune response (Tian et al., 2017). N protein interacts with viral RNA to form nucleocapsid and participate in virion assembly. GP5a protein, a newly discovered novel structural protein in arteritis, is a non-glycosylated membrane protein and is also essential for viral activity (Veit et al., 2014), but its function needs further study.

GP5 is a highly glycosylated capsule protein of about 25 kDa, with the highest degree of variation in PRRSV. It consists of a cleavable signal peptide at the N-terminal, an extracellular domain containing multiple N-glycoylation sites, three putative hydrophobic transmembrane domains, and a long hydrophilic cytoplasmic tail region (Veit et al., 2014). GP5 is a key protein in the assembly of viral particles and is involved in the pathogenesis of viruses. It is also a key target protein of neutralizing antibodies and can induce both cellular and humoral immune responses (Veit et al., 2014). M is a non-glycoylated protein of about 18–19 kDa and is one of the most conserved structural proteins of PRRSV. It consists of a short extracellular domain (15–17 aa), three putative hydrophobic transmembrane domains, and a long hydrophilic cytoplasmic tail region (Veit et al., 2014). The transport of GP5 and M from the ER to the Golgi apparatus requires heterodimerization of M with GP5, suggesting that only properly assembled GP5/M complexes can pass through the quality control system of the ER. At the same time, GP5/M heterodimers are also integrated into the virions and play an important role in the adsorption, assembly and budding processes, and essential for the formation of infectious virions.

An atypical PRRSV strain causing high mortality and abortion storms was emerged in the late 1990s initially in the United States, and subsequently in other areas (Mengeling et al., 1998). Since June 2006, the epidemic of “high fever,” caused by PRRSV variants, has caused great losses to the pig industry (Tian et al., 2007). Due to their high pathogenicity and virulence, these variants are also known as highly pathogenic PRRSV (HP-PRRSV; Xiao et al., 2008). Efforts were made to dissect the viral elements attributed to the high pathogenicity and virulence of these variants. Sequence comparison showed that many HP-PRRSV strains had a discontinuous deletion of several amino acids in nsp2, a multifunctional protein involved in viral replication and antiviral innate immune response (Allende et al., 2000; Kwon et al., 2008; Zhou et al., 2009; Faaberg et al., 2010; Morgan et al., 2013). A highly pathogenic PRRSV strain, HUB2, isolated from a pig farm in Hubei province also showed amino acid deletion in nsp2. So far, however, the association of nsp2 and other viral proteins from HP-PRRSV strains with their highly pathogenic phenotypes remains to be firmly established (Tong et al., 2007).

Regulation of the induction of antiviral and proinflammatory/inflammatory cytokines and chemokines is an important mechanism controlling the pathogenicity of many viruses. Indeed, secretion of several important cytokines (interleukin (IL)-8, IL-1β, interferon (IFN)-γ) is correlated with virus level, accounting for approximately 84% of the variations observed (Lunney et al., 2010). PRRSV infection induces the production of a variety of cytokines and inflammatory factors, including IL-1 and tumor necrosis factor-α (TNFα; Shen et al., 2000). PRRSV infection was reported to suppress host immune response by regulating IL-10 expression, resulting in increased mRNA levels of IL-1β, IFNα, IL-10, IL-12, TNFα and IFNγ during the first week of infection (Suradhat et al., 2003; Weesendorp et al., 2014). On the other hand, the quantity of innate cytokines secreted in PRRSV-infected pigs is significantly lower than with other viral infections (van Reeth et al., 1999). PRRSV mainly inhibited the secretion of type 1 IFNs (mainly IFN-a and IFN-β) by macrophages, and further inhibited the expression of antiviral factors induced by type 1 IFNs. It also stimulates Th1 cell-mediated immune responses that promote IFN-y expression by PBMC at 4–8 weeks after PRRSV infection (Meier et al., 2003).

We have previously reported the complete nucleotide sequence of PRRSV-SP, a vaccine strain with low pathogenicity (Shen et al., 2000). In this study, a recombinant PRRSV-SP, PRRSV-rSP, was initially rescued from the full-length cDNA clone of PRRSV-SP. By replacing ORF5 and ORF6, coding for GP5 and M proteins, respectively, with the corresponding regions from the highly pathogenic strain PRRSV-HUB2, a chimeric recombinant virus, rSP-HUB2 was subsequently rescued. The two recombinant viruses were found to be genetically stable and share similar growth kinetics, with rSP-HUB2 showing apparent growth and fitness advantages. Interestingly, the two viruses induced differential expression of a number of antiviral and proinflammatory cytokines and chemokines. Further overexpression, deletion and mutagenesis studies demonstrated that amino acid residue F16 in the N-terminal region of the GP5 protein from HUB2 was a determinant for the phenotypic difference between the two recombinant viruses. This study provides evidence suggesting that GP5 may function as a potential determinant for the pathogenicity and virulence of HP-PRRSV.

## Materials and methods

### Virus, cell line, antibodies and reagents

PRRSV vaccine strain SP GenBank: (AF184212.1) was originally obtained from the Schering-Plough Animal Health Company and sequences for the GP5 and M from the highly pathogenic strain HUB2 were synthesized based on GenBank: EF112446.1.

PAM cells were prepared from 35-day-old normal weaned piglets (with PRSSV and anti-PRRSV antibodies testing negative) by removing the whole lungs and injecting 50 ~ 100 mL sterilized PBS into the lungs from the trachea. After gently patting the surface of the lungs and gently rubbing repeatedly for 2–3 min, the lavage solution was recovered, filtered and centrifuged. The precipitated cells were re-suspended in RPMI 1640 nutrient medium with 10% FBS and cultured at 37°C, 5% CO_2_. Marc-145 cell line was purchased from the American Type Culture Collection (ATCC10031), and IPAM cells (HTX2097) was purchased from Otwo Biotech (ShenZhen) Inc.

PRRSV positive serum was provided by Huanong (Zhaoqing) Biological Industry Technology Research Institute, China; HRP conjugated Goat anti-pig IgG was purchased from (Earthox); TIANprep Mini Plasmid Kit, FastKing RT Kit (with gDNase) and SuperReal PreMix Plus (SYBR Green) were purchased from Tiangen Biochemical Science and Technology; Gel Extraction Kit from OMEGA; Reverse Transcriptase M-MLV(RNase H-), 5 × Reverse Transcriptase M-MLV Buffer, RNase Inhibitor and Random primers from TaKaRa; DMEM, FBS, pancreatin, penicillin and streptomycin from Gibco; restriction enzymes and high concentration T4 DNA Ligase from NEB; TransZol, 2 × EasyPfu PCR SuperMix(−dye), 2 × EasyTaq^®^ PCR SuperMix(+dye), pEASY^®^-Basic Seamless Cloning and Assembly Kit from TransGen Biotech; mMESSAGE mMACHINE™ T7 Transcription Kit from Ambion; AEC(IHC) chromogenic working solution from Thermo.

### Plasmids construction

The full-length cDNA clone of pBR322-PRRSV-SP was constructed by inserting the PRRSV-SP full-length cDNA into pBR322 under the control of the T7 promoter, followed by the T7 terminator and HDV sequence to ensure an accurate and productive mRNA synthesis. To distinguish the recombinant virus from WT virus, a T to A mutation at nucleotide position 3,448 was purposely introduced without altering the original amino acid sequence but eliminated a BsmBI site at the position. To construct pBR322-PRRSV-SP/HUB2, one fragment containing GP5 and M genes from HUB2 strain and three DNA fragments covering other regions of PRRSV-SP were joined by homologous recombination with the pEASY^®^-Basic Seamless Cloning and Assembly Kit.

Plasmids XJ40-GP5(SP), XJ40-M(SP), XJ40-GP5(HUB2) and XJ40-M(HUB2) were constructed by cloning the viral sequences into pXJ40 with the homologous recombination technique. Plasmid XJ40-GP5(HUB2/SP) was constructed by replacing the N-terminal 60 amino acids of GP5 protein from SP strain with the equivalent region from HUB2 strain; pXJ40-GP5(HUB2/SP)-M1, pXJ40-GP5(HUB2/SP)-M2 and pXJ40-GP5(HUB2/SP)-M3 were constructed by introducing F16 to S, Y24 to C and NNN33-35 to YSS mutations, respectively, with the homologous recombination technique.

The templates and primers used for construction of these plasmids were listed in Table 1.

**Table 1:** Templates and primers for plasmids construction.

Plasmid	Template	Primer (5′–3′)
pXJ40-GP5(SP)	pBR322-PRRSV-SP	Fwd: ACGCGGATCCATGTTGGGGAAATGCTTGACC Rev.: ACCGCTCGAGCTAGGGACGACCCCATTGTTC
pXJ40-M(SP)		
pXJ40-GP5(HUB2)	pBR322-PRRSV-SP/HUB2	Fwd: GATGATAAGTCCGGATCCATGTTGGGGAAGTGCTGAC Rev.: CTAGAGACGACCCCATTGTTCC
pXJ40-M(HUB2)		
pXJ40-GP5(HUB2/SP)	pXJ40-GP5(SP) pXJ40-GP5(HUB2)	Fwd: GATGATAAGTCCGGATCCATGTTGGGGAAGTGCTTGAC Rev.: CTAGAGACGACCCCATTGTTCC
pXJ40-GP5(HUB2/SP)-M1	pXJ40-GP5(HUB2/SP)	Fwd: CGATTGCTTTCTTTGTGGTGTATC Rev.: CCTGTTTTTGCTCACCCAGAAA Fwd: GGTGAGCAAAAACAGGAAGGCAA Rev.: CCACAAAGAAAGCAATCGCGAGCAAC
pXJ40-GP5(HUB2/SP)-M2		Fwd: ATCGTGCCGTTCTGTCTTGCTG Rev.: CCTGTTTTTGCTCACCCAGAAA Fwd: GGTGAGCAAAAACAGGAAGGCAA Rev.: ACAGAACGGCACGATACACCAC
pXJ40-GP5(HUB2/SP)-M3		Fwd: CTCGTCAACGCCAGCTACAGCAGCAGCTCTCATATTCAG Rev.: CCTGTTTTTGCTCACCCAGAAA Fwd: GGTGAGCAAAAACAGGAAGGCAA Rev.: GTAGCTGGCGTTGACGAGCAC

### Rescue of recombinant PRRSVs

The full-length plasmid BR322-PRRSV-SP and BR322-PRRSV-SP/HUB2 were linearized with NotI, purified with phenol/chloroform/isoamyl alcohol (25,24, 1), and used for *in vitro* transcription in 20 μL standard reaction volume according to the manufacturer’s instruction. These transcripts were transfected into Marc-145 cells by electroporation. After incubating for 4–5 days until cytopathic lesions appeared, cells were harvested by freeze–thaw method and centrifuged at 5,000 rpm for 10 min at 4°C. The supernatants were harvested as the P0 virus stocks and stored at − 80°C for future usage.

### Validation of the rescued PRRSV-rSP and rSP-HUB2 by immunocytochemistry, Weston blot and sequencing

Each 100 μL of 10-time diluted recombinant virus from the viral stocks was inoculated into 96-well plates of MARC-145 monolayer cells. After adsorption at 37°C for 1 h, the medium was replaced with fresh DMEM containing 2% FBS. After incubation for 48 h, cells were washed twice with phosphate buffer and permeabilized with pre-cooled methanol at – 20°C for 30 min, incubated with anti-PRRSV serum and HRP conjugated goat anti-pig IgG with standard method.

Marc-145 cells were infected with the recombinant virus for 48–72 h and harvested. Cells were lysed with 150 μL of RIPA Buffer containing 1 mM PMSF, and analyzed by Western blot with anti-PPRSV serum and HRP conjugated goat anti-pig IgG. The protein expression was visualized with an Azure Biosystems C600 imaging system.

For sequencing confirmation, P0 virus stocks were passaged in MARC145 cells for 5–20 generations, and total RNAs were extracted for RT-PCR amplification and sequencing.

### Characterization of the recombinant virus

Plaque assay, TCID50 and one-step growth curves were generally performed with standard methods in Marc-145 cells. Briefly, TCID50 was determined calculation with the Reed-Muench method by immunocytochemistry; for determining the one-step growth curves of PRRSV-rSP and rSP-HUB2, cells were infected with each virus at the multiplicity of infection (MOI) of 0.1, harvested at 0, 12, 24, 36, 48, 60, and 72 h post-infection (hpi), and TCID50 for each sample was determined. The average titers were calculated from triplicates.

### RT-qPCR determination of viral and cellular RNAs

Total RNAs from virus-infected or transfected cells were extracted by the Trizol method and used as templates for the RT reaction with the FastKing gDNA dispelling RT superMix system (Tiangen). In a 20 μL standard reaction, 2 μg of template RNA was mixed with 4 μL of 5 × Fastking-RT SuperMix (including FastKing RT enzyme, RNase inhibitor, random primers, oligo-dT primer, dNTP mixture and reaction buffer), supplemented with RNase-free water. The reaction was performed at 42°C for 15 min and 95°C for 3 min, and cDNA products were diluted 20-fold for qPCR analysis following the instructions for Super Real PreMix Plus (SYBR Green; Tiangen). In a 20 μL standard reaction, 8.4 μL of diluted cDNA, 10 μL of 2 × SuperReal PreMix, 0.4 μL of 50 × ROX reference dye, 0.6 μL of 10 μM forward primer (F) and 0.6 μL of 10 μM reverse primer (R) were added. The reaction procedure was set as follows: enzyme activation at 50°C for 2 min, pre-denaturation at 95°C for 3 min, denaturation at 95°C for 3 s, annealing and extension at 60°C for 15 s (40 cycles). The melting curve was analyzed at 95°C for 15 s and 60°C for 60 s, and the results were presented as cyclic threshold (CT) values. The 2^–△△CT^ method was used to calculate the data and analyze the gene expression level with GAPDH as the reference gene.

### Statistical analysis

The one-way ANOVA method was used to analyze the significant difference between the indicated sample and the respective control sample. Significance levels were presented by the value of p (ns, non-significant; ^*^p < 0.05; ^**^p < 0.01; ^****^p < 0.0001).

## Result

### Recovery of PRRSV-rSP vaccine strain and chimeric rSP-HUB2 strain from full-length cDNA clones

**Figure 1:**
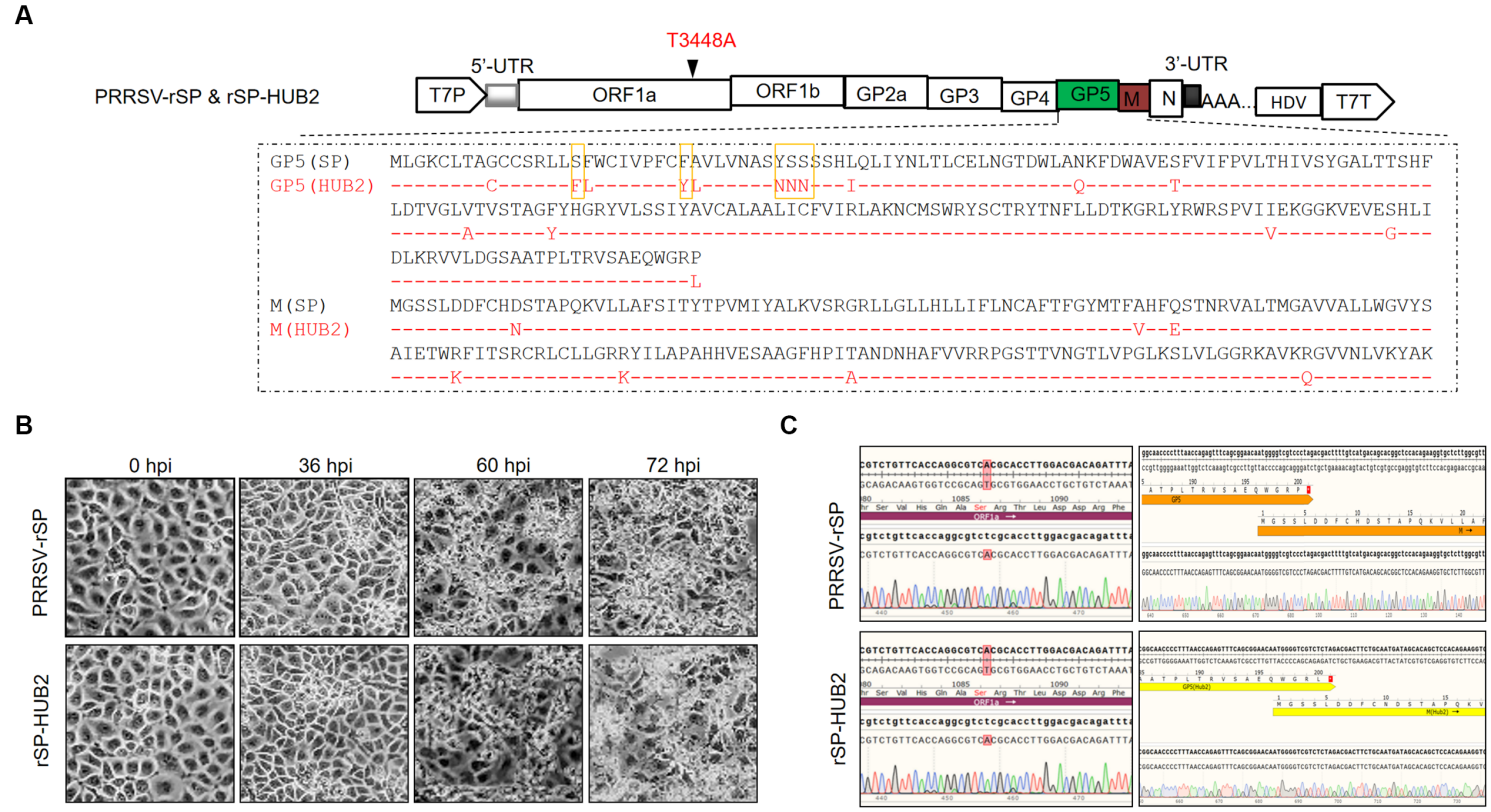
Rescue of recombinant PRRSV-rSP strain and chimeric rSP-HUB2 strain. **(A)** Diagram showing the genomic organization of PRRSV-rSP and rSP-HUB2. Illustrated include the positions of the T7 promoter, 5′-untranslated region (5′-UTR), different ORFs and protein-coding regions, the T3448A mutation, 3′-UTR, hepatitis delta virus sequence and the T7-terminator. The sequence comparison of GP5 and M proteins between the two recombinant viruses and the five boxed amino acids in GP5 used for mutagenesis in this study are also indicated. **(B)** Cytopathic effects of Marc145 cells transfected with the full-length *in vitro* transcribed PRRSV-rSP and rSP-HUB2 RNAs in time course experiments. The *in vitro* transcribed RNAs were electroporated into Marc145 cells and phase images were taken at 0, 24, 36, 48, 60 and 72 h post-electroporation, respectively. **(C)** Sequence validation of PRRSV-rSP and rSP-HUB2. The regions covering the T3448A mutation and the junction between GP5 and M were sequenced and shown.

To recover a recombinant virus from the SP vaccine strain of PRRSV, the full-length cDNA was cloned into a pBR322-based plasmid flanked by the T7 promoter and T7 terminator sequences, generating pBR322-PRRSV-rSP (Figure 1A). Transfection of Marc145 cells with full-length *in vitro* transcripts from pBR322-PRRSV-rSP resulted in the rescue of the recombinant virus PRRSV-rSP. To recover a chimeric virus with GP5 and M genes replaced by equivalent sequences from a highly pathogenic strain, PRRSV-HUB2, pBR322-rSP-HUB2(GP5M) was constructed (Figure 1A). Transfection of Marc145 cells with the full-length *in vitro* transcripts from this construct led to the rescue of recombinant virus rSP-HUB2. In both cases, obvious pathogenic changes were observed in Marc145 cells from 36 h post-transfection (Figure 1B). The successful rescue of these two recombinant viruses was further confirmed by sequence verification, showing a point mutation (T3448A) and HUB2 strain-specific sequences in rSP-HUB2, and wild type SP sequence in PRRSV-rSP (Figure 1C). It was also noted that cytopathic changes usually appeared a few hours earlier in cells transfected with rSP-HUB2 transcripts than did in cells transfected with PRRSV-rSP transcripts.

### Characterization of the growth properties and stability of PRRSV-rSP and rSP-HUB2

**Figure 2:**
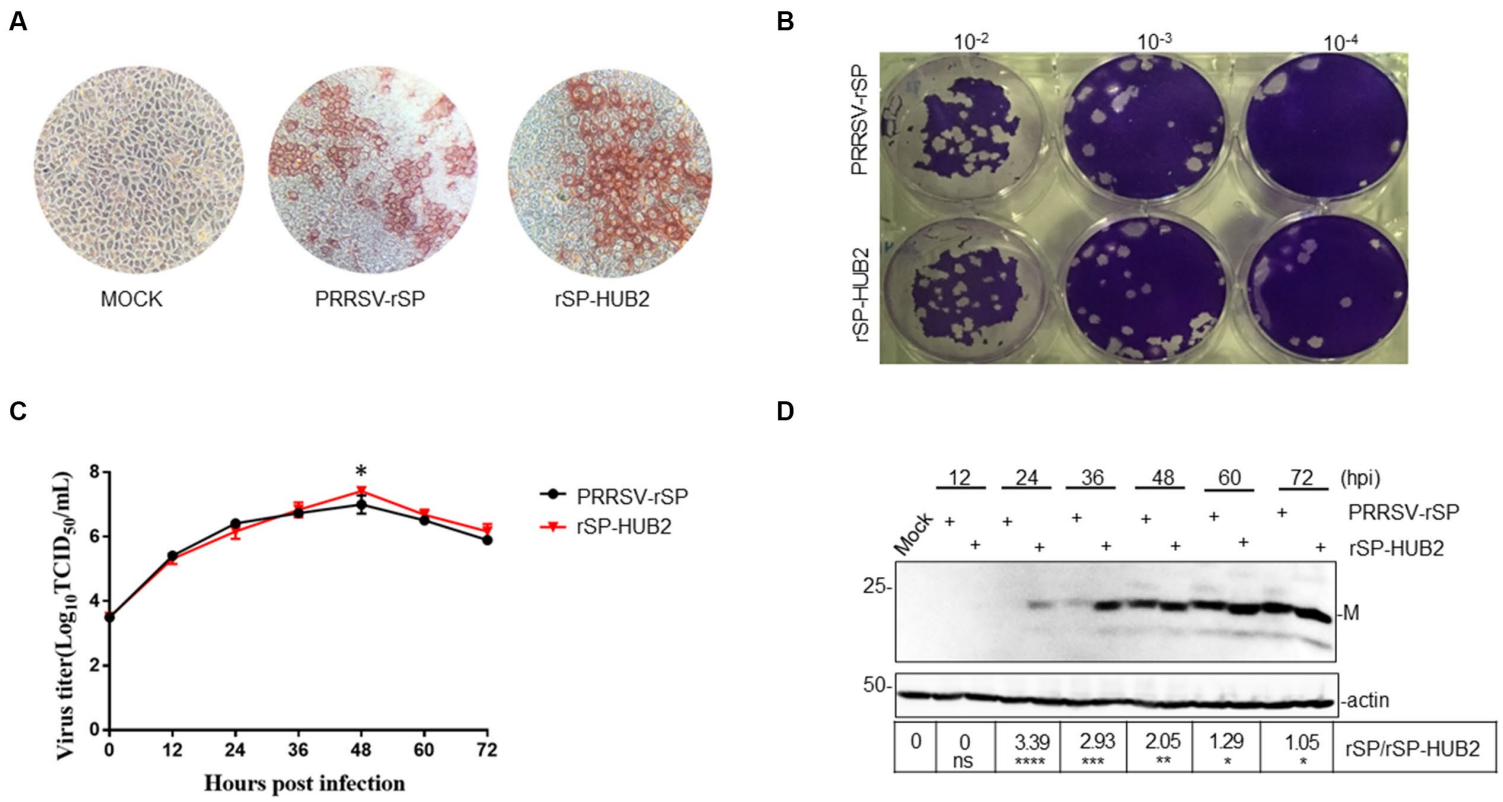
Characterization of the growth properties of PRRSV-rSP and rSP-HUB2. **(A)** Immunocytochemical examination of cells infected with PRRSV-rSP and rSP-HUB2. The recovered viruses were passaged in Marc-145 cells for 5 passages, harvested and 10-serially diluted. 100 μL each well of the diluted viruses were added to 96-well plates of Marc-145 cells with 90% confluency, cultured for 72 h, stained by immunocytochemistry and examined by light microscopy (20 ×). **(B)** Plaque assay. 200 μL each well of the 10-serially diluted viruses were added to Marc-145 cells in six-well plates. After incubation for 2 h, 2.5 mL of DMEM media mixed with low-melted-agarose were added to each well, continued incubation for 2–3 days, and visualized by staining with crystal violet. **(C)** Growth kinetics. Monolayers of Marc-145 cells were infected with PRRSV-rSP and rSP-HUB2, respectively, at an MOI of 0.1, and harvested at different time points post-infection. The titers of each virus were determined by TCID50 and plotted. ^*^p < 0.05. **(D)** Western blot analysis of viral M protein in Marc-145 cells infected with PRRSV-rSP and rSP-HUB2. Marc-145 cells were infected with PRRSV-SP and rSP-HUB2, respectively, at an MOI of 0.1. Protein samples were resolved by SDS-PAGE and analyzed Western blot using a specific polyclonal antibody against M protein. Beta-actin was used as the loading control. Numbers on the left indicate protein sizes in kilodalton.

The growth properties of PRRSV-rSP and rSP-HUB2 in Marc145 cells were first characterized by immunocytochemistry and plaque assay. Similar shapes and sizes of the infection foci appeared in cells infected with the two viruses (Figure 2A), but slightly smaller plaques and higher viral titers were observed in rSP-HUB2-infected cells (Figure 2B). Further determination of the growth curves by TCID50 also revealed similar growth kinetics of the two recombinant viruses (Figure 2C). Once again, rSP-HUB2 reached a moderately higher (0.5 log) peak titer at 48 hpi, compared with the peak titer of PRRSV-rSP at the same time point post-infection (Figure 2C). Determination of viral protein expression by Western blot confirmed that earlier and moderately, but significantly higher accumulation of M protein was detected in cells infected with rSP-HUB2, compared with that in cells infected with PRRSV-rSP (Figure 2D). Certain inconsistencies between viral titers and the protein levels at various time points post-infection in these and several other repeated experiments were noted (Figures 2C,D). These inconsistencies might be caused by the relatively low sensitivity and experimental variations of the TCID50 assay.

The genetic stability of the two viruses was determined by nucleotide sequencing after passage in Marc145 cells for 22 passages, confirming that no additional nucleotide mutations occurred in the two recombinant viruses during these passages. Taken together, these results indicate that replacement of GP5 and M in PRRSV-rSP with the equivalent sequences from the highly pathogenic HUB2 strain may render a growth advantage to the vaccine strain in Marc145 cells.

### Differential induction of several innate immune and inflammatory genes in Marc145, IPAM and PAM cells infected with PRRSV-rSP and rSP-HUB2

**Figure 3:**
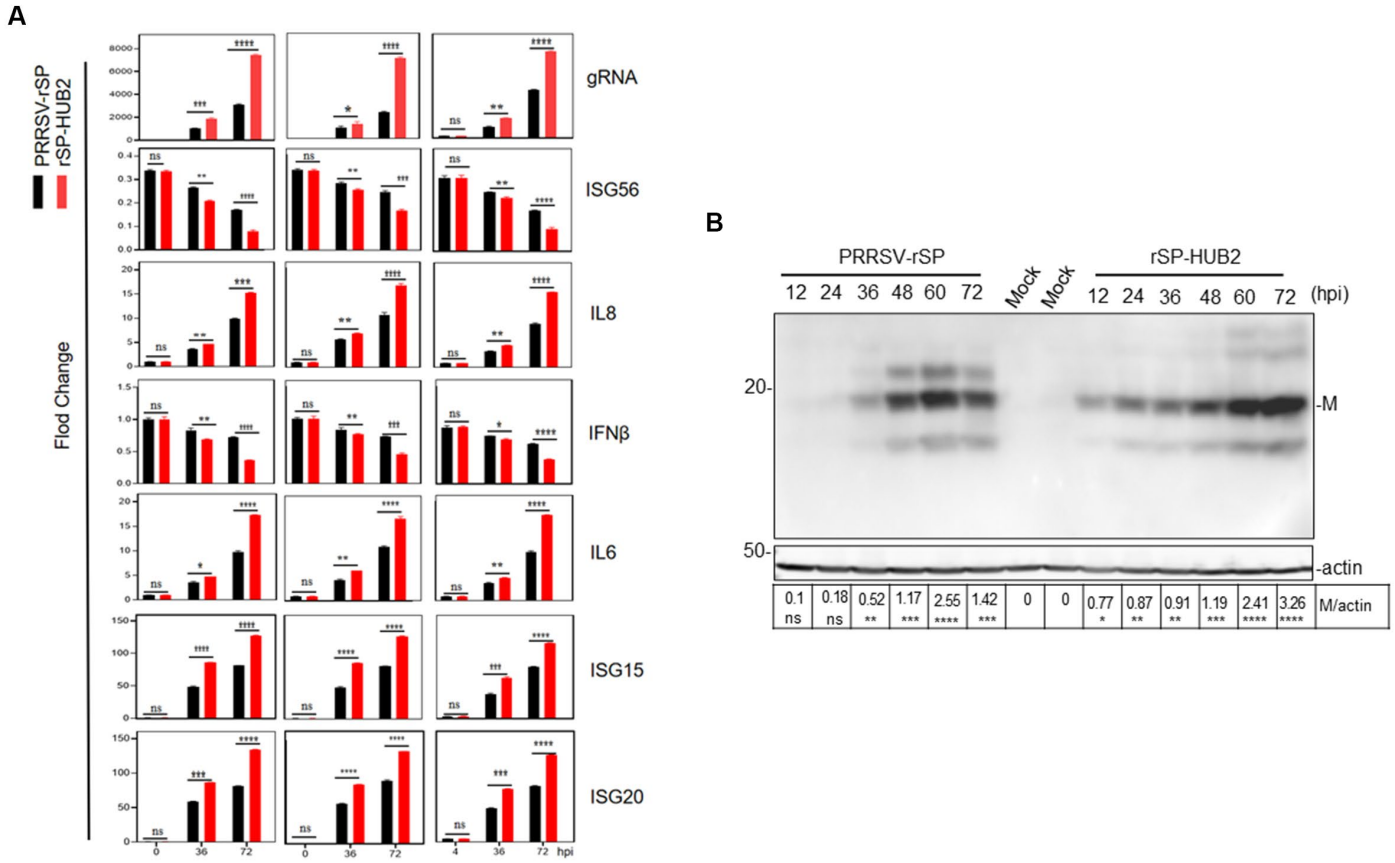
Differential induction of cytokines in Marc-145 and PAM cells infected with PRRSV-rSP and rSP-HUB2, as well as in IPAM cells transfected with the two viral RNAs, respectively. **(A)** RT-qPCR analysis of viral gRNA and mRNA levels of IL6, IL8, ISG15, ISG20, IFN-β and ISG56 in Marc-145, PAM and IPAM cells infected with PRRSV-rSP and rSP-HUB2, respectively. Cells were separately infected with PRRSV-rSP and rSP-HUB2 at an MOI of 1, harvested at 0, 36 and 72 hpi, respectively. Total RNAs were extracted and the levels of viral gRNA and above cytokines/chemokines were determined by RT-qPCR.^*^p < 0.05; ^**^p < 0.001; ^***^p < 0.0001; ****p < 0.00001. **(B)** Western blot analysis of viral M protein. Cells were infected as described in **(A)** and viral M protein was analyzed by Western blot.

To determine if the differences in the growth kinetics between the two recombinant viruses were dictated by their differential ability to induce cellular innate immune and inflammatory responses, Marc-145 and PMA cells were infected with 0.1 MOI of PRRSV-rSP and rSP-HUB2, and harvested at 0, 4, 12, 24, 36, 48, 60 and 72 hpi, respectively, for total RNA extraction and real-time qPCR analysis. The results demonstrated that the expression levels of IL-6, IL-8, IFN-β, ISG15, ISG20 and ISG56 were indeed differentially upregulated. As shown in Figure 3A, significantly higher induction levels of IL-6, IL-8, ISG15 and ISG20, respectively, were detected in rSP-HUB2-infected cells at 36 and 72 hpi, compared to the induction levels of these factors in cells infected with PRRSV-rSP at the same time points. On the contrary, the induction levels of IFN-β and ISG56 were more dramatically reduced in cells infected with rSP-HUB2 at 36 and 72 hpi, compared to their levels in cells infected with PRRSV-rSP at the same time points (Figure 3A). A similar differential induction pattern of these genes was also observed in these cells infected with the two recombinant viruses at other time points (Supplementary Figure 1). Consistently, the viral genomic RNA (gRNA) level of rSP-HUB2 was significantly higher than PRRSV-rSP (Figure 3A).

Western blot analysis of Marc-145 cells infected with the two recombinant viruses confirmed that earlier and generally higher accumulation of M protein was detected in cells infected with rSP-HUB2, compared with that in cells infected with PRRSV-rSP (Figure 3B). Taken together, these results confirm the differential induction of innate immune and proinflammatory responses by the two recombinant viruses, and prompted the subsequent studies to determine the roles played by the replacement region in chimeric rSP-HUB2 in the induction of these cytokines and chemokines.

### Identification of GP5 protein from the highly pathogenic HUB2 strain as a main determinant responsible for the differential induction of innate immune and inflammatory responses

**Figure 4:**
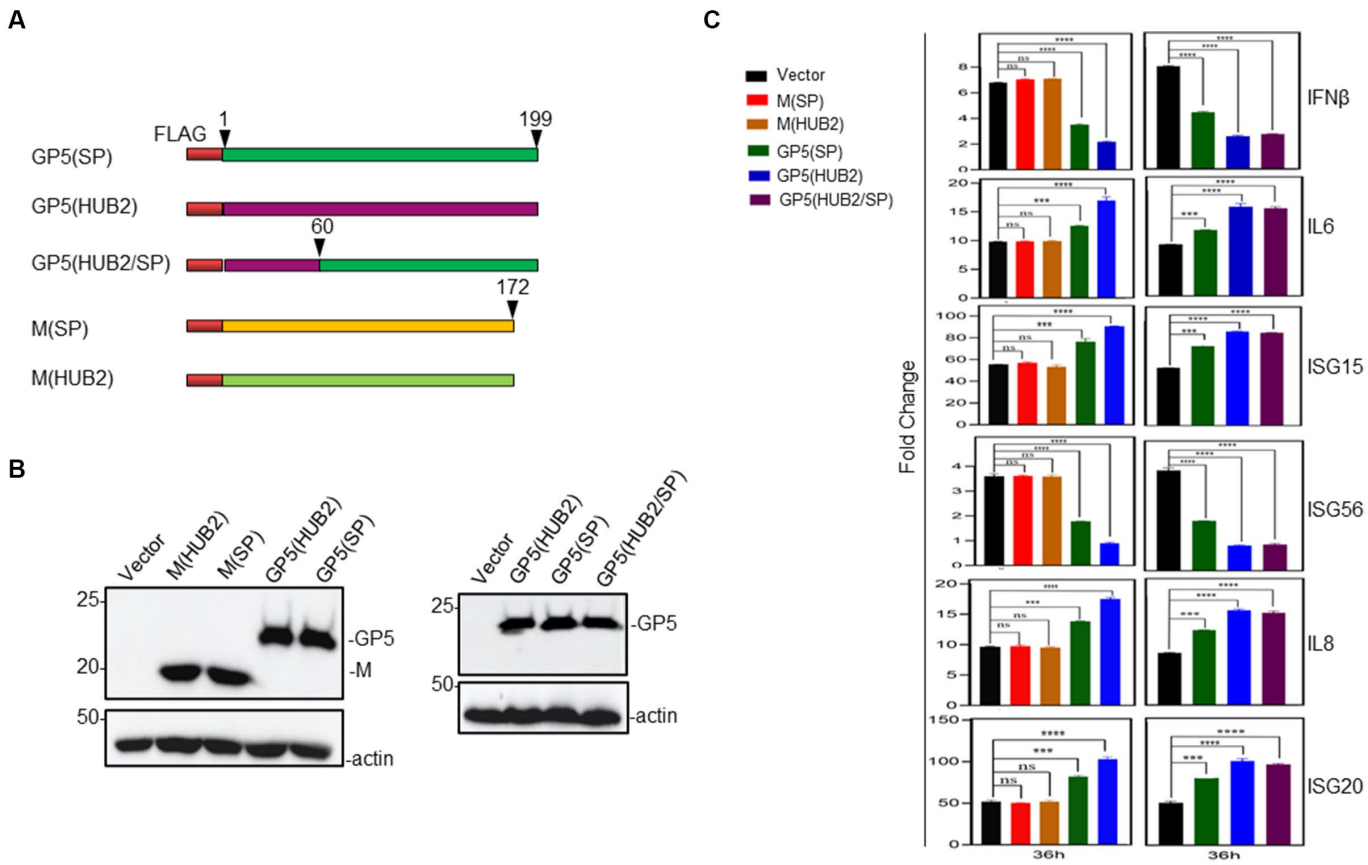
Effects of overexpression of GP5 and M proteins on Poly(I:C)-induced expression of IL-6, IL-8, ISG15, ISG20, IFN-β and ISG56 in MARC-145 cells. **(A)** Diagram showing three GP5 and two M constructs used in the overexpression experiments. The regions of GP5 and M from two different viruses were shown in different colors. **(B)** Western blot analysis of the expression of M and GP5 proteins in transfected cells. Marc-145 cells were transfected pXJ40-FLAG-M(HUB2), pXJ40-FLAG-M(SP), pXJ40-FLAG-GP5(HUB2), pXJ40-FLAG-GP5(SP) and empty vector pXJ40-FLAG, respectively, together with Poly(I:C), and harvested at 48 h post-transfection. Protein samples were resolved by SDS-PAGE and analyzed by Western blot using an anti-FLAG antibody. Beta-actin was used as the loading control. Numbers on the left indicate protein sizes in kilodalton. **(C)** RT-qPCR analysis of mRNA levels of IL-6, IL-8, ISG15, ISG20, IFN-β and ISG56 in transfected Marc-145 cells. Cells were transfected as described in **(B)** and harvested at 36 h points post-transfection. Total RNAs were extracted and mRNA levels of above cytokines/chemokines were determined by RT-qPCR.^*^p < 0.05; ^**^p < 0.001; ^***^p < 0.0001; ^****^p < 0.00001.

In order to identify the viral component(s) responsible for the differential induction of the innate immune and inflammatory genes, overexpression of M and GP5 proteins from PRRSV-rSP and rSP-HUB2, respectively, was carried out. The induction of the expression of these innate immune and inflammatory response-related genes was stimulated by Poly(I:C) instead of PRRSV infection to avoid the effects rendered by the viral proteins produced during viral replication. Marc145 cells were co-transfected with pXJ40-FLAG-M(HUB2), pXJ40-FLAG-M(SP), pXJ40-FLAG-GP5(HUB2), pXJ40-FLAG-GP5(SP) or the empty vector pXJ40-FLAG, respectively with Poly(I:C; Figure 4A). The mRNA levels of IL-6, IL-8, IFN-β, ISG15, ISG20 and ISG56 at 12, 24, 36 and 48 h post-transfection were determined by RT-qPCR. Analysis of the M and GP5 protein levels by Western blot showed the expression of these proteins at very similar levels (Figure 4B). Overexpression of M protein from the two viruses did not render significant differential effects on the induction of these genes (Figure 4C). In cells overexpressing GP5 protein from both SP and HUB2 strains, however, differential expression of these cytokines and chemokines was observed. As shown in Figure 4C, overexpression of GP5(HUB2) induced significantly lower expression levels of IFN-β and ISG56, but significantly higher expression levels of IL-6, IL-8, ISG-15 and ISG-20, respectively, compared to the expression levels of these factors in cells transfected with GP5 from PRRSV-rSP at 36 h post-transfection. A similar pattern of differential induction of these genes in cells overexpressing the two GP5 proteins was also observed at other time points (Supplementary Figure 2). These results demonstrate that the GP5 protein from the highly pathogenic HUB2 strain may be the main determinant for the differential induction of these genes.

To further confirm and to locate the functional motif in GP5 responsible for this differential induction, the amino acid sequences of the two GP5 genes were compared, revealing that the major sequence differences were in the N-terminal first 60-amino-acid-region between the two GP5 proteins (Figure 1A). Accordingly, the first 60 amino acids in GP5(SP) were replaced with the equivalent region from GP5(HUB2), generating an expression plasmid pXJ40-FLAG-GP5(HUB2/SP) expressing the chimeric GP5 protein GP5(HUB2/SP; Figure 4A). Transfection of Marc145 cells with pXJ40-FLAG-GP5(HUB2/SP), pXJ40-FLAG-GP5(SP), pXJ40-FLAG-GP5(HUB2) and pXJ40-FLAG, respectively, in the presence of Poly(I:C) demonstrated that expression of the chimeric GP5 (GP5(HUB2/SP)) and GP5(HUB2) produced a similar enhancement effect on the induction of IL-6, IL-8, ISG15 and ISG20 and a similar suppressive effect on the expression of IFN-β and ISG56 (Figures 4B,C; Supplementary Figure 2). These results indicate that the sequence differences in the first 60 amino acids between GP5(SP) and GP5(HUB2) may play an essential role in the differential induction of these genes.

### Identification of amino acid residues in GP5 protein critical for the differential induction of innate immune and proinflammatory responses

**Figure 5:**
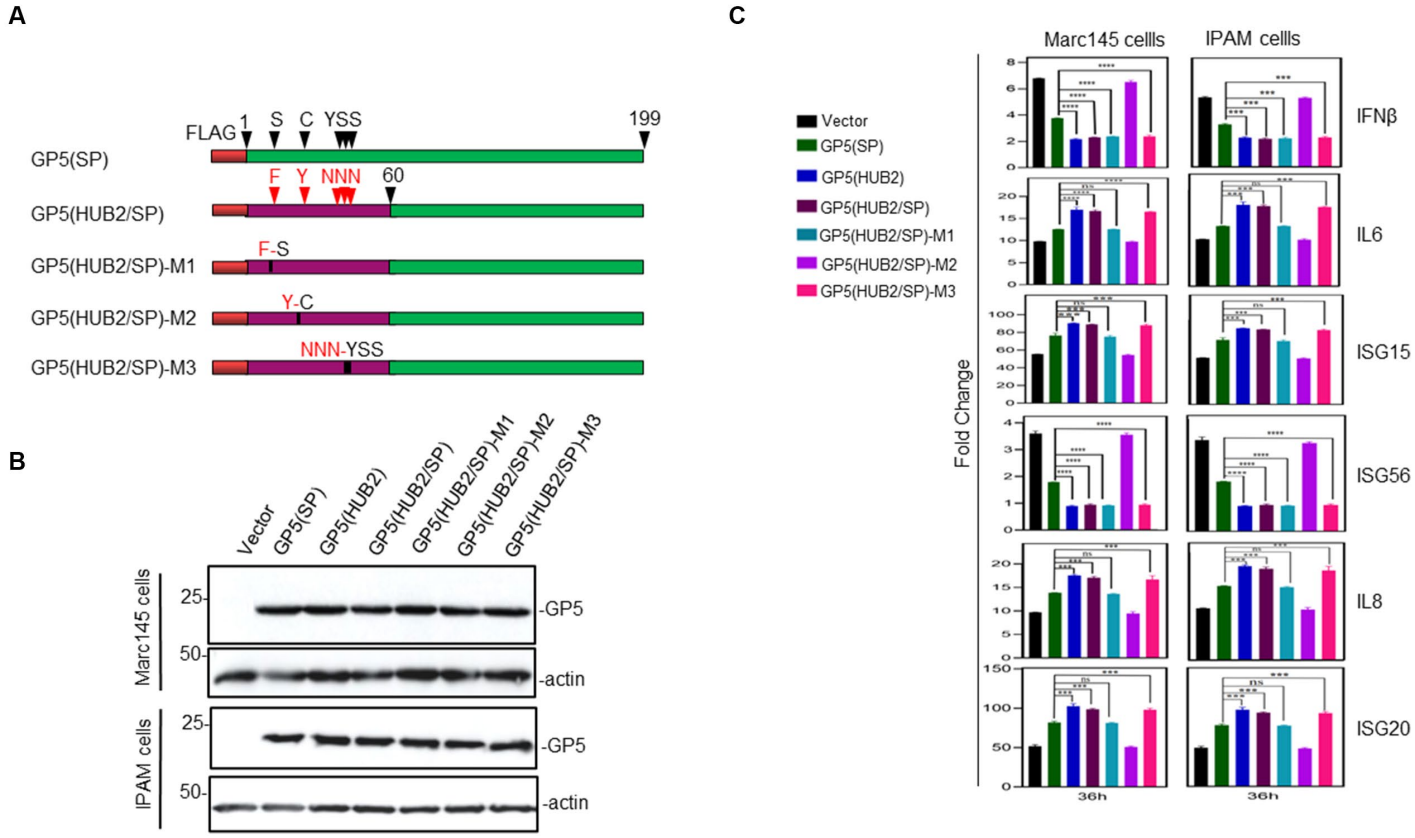
Effects of GP5-HUB2F16S/SP, GP5-HUB2Y24C/SP and GP5-HUB2NNN33–35YSS/SP on IL-6, IL-8, ISG15, ISG20, IFN-β and ISG56 expressions triggered by poly(I:C) in Marc-145 and IPAM cells. **(A)** Diagram showing five wild type and mutant GP5 constructs used in the overexpression experiments. The regions of GP5 from two different viruses and point mutations introduced were illustrated. **(B)** Western blot analysis of the expression of M and GP5 proteins in transfected cells. Marc-145 and IPAM cells were transfected pXJ40-FLAG-GP5-HUB2F16S/SP, pXJ40-FLAG-GP5-HUB2Y24C/SP, pXJ40-FLAG-GP5-HUB2NNN33–35YSS/SP, pXJ40-FLAG-GP5-HUB2, pXJ40-FLAG-GP5(HUB2/SP), pXJ40-FLAG-GP5-SP and empty vector pXJ40-FLAG, respectively, together with Poly(I:C), and harvested at 48 h post-transfection. Protein samples were resolved by SDS-PAGE and analyzed by Western blot using an anti-FLAG antibody. Beta-actin was used as the loading control. Numbers on the left indicate protein sizes in kilodalton. **(C)** RT-qPCR analysis of mRNA levels of IL-6, IL-8, ISG15, ISG20, IFN-β and ISG56 in transfected Marc-145 and IPAM cells. Cells were transfected as described in **(B)** and harvested at 36 h post-transfection. Total RNAs were extracted and mRNA levels of above cytokines/chemokines were determined by RT-qPCR. ^*^p < 0.05; ^**^p < 0.001; ^***^p < 0.0001; ^****^p < 0.00001.

Comparison of the first 60 amino acid sequences between the two proteins showed differences at 10 amino acid positions between GP5(HUB2) and GP5(SP; Figure 1A). Five amino acids (F16S, Y24C, N33Y, N34S, and N35S) with significant changes in the amino acid properties were mutated back to the equivalent sequences in GP5(SP) individually or with three amino acids together (Figures 1A, 5A). Two single-point (F16S and Y24C) and one triple-point (N33Y, N34S and N35S) mutations were introduced into the GP5(HUB2/SP) sequence, generating pXJ40-FLAG-GP5(HUB2/SP)-M1 (contains the F16S mutation), pXJ40-FLAG-GP5(HUB2/SP)-M2(Y24C mutation) and pXJ40-FLAG-GP5(HUB2/SP)-M3 (N33Y, N34S and N35S mutations; Figure 5A). Very similar expression efficiencies of these mutant proteins were detected in both Marc-145 and IPAM cells transfected with these constructs (Figure 5B).

Transfection of Marc145 and IPAM cells with pXJ40-flagGP5(HUB2/SP)-M3 in the presence of Poly(I:C) showed a similarly differential induction pattern of IL-6, IL-8, ISG15, ISG20, IFN-β and ISG56 as in cells overexpressing GP5(HUB2) and GP5(HUB2/SP), respectively, at 36 h post-transfection (Figure 5C), ruling out the potential involvement of these three residues in the differential induction of these genes (Figure 5C). Transfection of Marc145 and IPAM cells with pXJ40-flagGP5(HUB2/SP)-M1 in the presence of Poly(I:C) showed a similar induction pattern of IL-6, IL-8, ISG15 and ISG20 as in cells overexpressing GP5(SP), but the induction pattern of IFN-β and ISG56 was more similar to that in cells transfected with GP5(HUB2) and GP5(HUB2/SP), respectively, at 36 h post-transfection (Figure 5C). Very similar induction patterns of these genes were also observed in both Marc145 and IPAM cells overexpressing the two mutant constructs at other time points post-transfection (Supplementary Figure 3). These results suggest that amino acid residue Y16 might be involved in the differential induction of these pathogenic factors, but not in the induction of the two IFN genes. For some unknown reasons, however, the expression of flagGP5(HUB2/SP)-M2 in both Marc145 and IPAM cells induced an identical induction profile as in cells transfected with the vector control (Figure 5C; Supplementary Figure 3). Taken together, these results demonstrate that the F16-containing motif would be responsible for the differential induction of these proinflammatory genes by the two GP5 proteins.

### The growth and fitness advantage of rSP-HUB2

**Figure 6:**
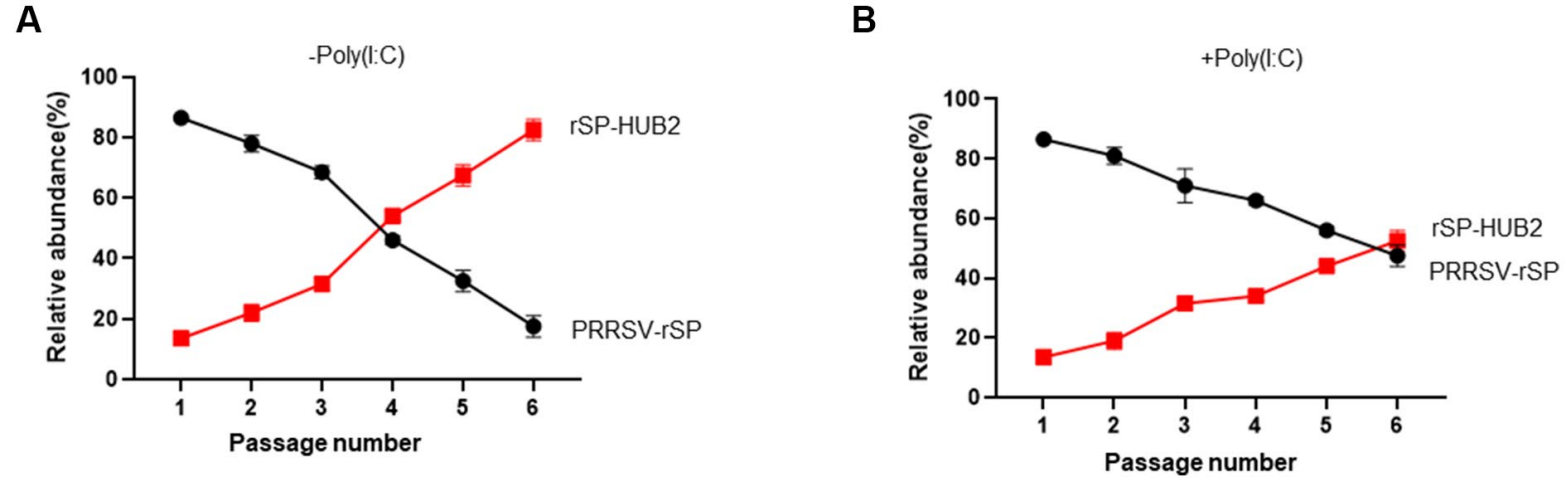
The growth and fitness advantages of rSP-HUB2 **(A)** Competition assays between rSP-HUB2 and PRRSV-rSP in the absence of type I IFN activation. Marc-145 cells were infected with PRRSV-rSP and rSP-HUB2 mixture at the ratio of 9:1 and continuously passaged for 6 passages. The relative abundance of each virus was determined by sequencing the GP5-M regions. **(B)** Competition assays between rSP-HUB2 and PRRSV-rSP in the presence of type I IFN activation. Marc-145 cells were transfected with Poly(I:C) and infected with PRRSV-rSP and rSP-HUB2 mixture at the ratio of 9:1 4 h post-transfection. After repeating the transfection/infection for 6 passages, the relative abundance of each virus was determined by sequencing the GP5-M regions.

The growth and fitness advantages of rSP-HUB2 over PRRSV-rSP were then tested by a competition assay in Marc-145 cells in the presence or absence of the type I IFN activation. Cells were treated with or without Poly(I:C), and infected with an MOI of approximately 0.1 of PRRSV-rSP and rSP-HUB2 mixture at the ratio of 9:1, and continuously passaged for 6 passages. Total RNAs were extracted from each passage, the GP5-M regions were amplified by RT-PCR, and the PCR products were sequenced to determine the relative abundances of the GP5M region from the two viruses, by comparing and calculating the average peaks between F16 and S16, Y24 and C24, and N33 and Y33. In the absence of type I IFN activation, the relative abundance of rSP-HUB2 clones were increased from 10 to 90% after 6 passages (Figure 6A). In the presence of Poly (I:C), the relative abundance of rSP-HUB2 clones were also increased, but at a slower pace (Figure 6B). It rose from 10% at passage 1 to 60% at passage 6 (Figure 6B). These results confirm that replacement of the GP5-M region in rSP-HUB2 indeed renders advantages in growth, fitness and evasion of type I IFN action to the recombinant virus, unraveling an important potential virulence determinant in this region.

## Discussion

The pathogenicity and virulence of different PRRSV strains vary dramatically, but the main virulence determinant(s) is yet to be firmly identified and characterized. In this study, a recombinant virus, rSP-HUB2, was rescued by replacing GP5 and M genes in vaccine strain PRRSV-rSP with equivalent sequences from a highly pathogenic strain HUB2, using a reverse genetics approach. The rescued rSP-HUB2 exhibited growth and fitness advantages in cultured cells and induced differential expression of a number of innate immune and proinflammatory genes. These include a significantly higher induction of IL-6, IL-8, ISG15 and ISG20, and a significantly more suppression of IFN-β and ISG56 induction, compared with its parental strain PRRSV-rSP. Further studies by overexpression, deletion and mutagenesis revealed that F16 at the N-terminal first 60 amino acids of GP5 may play an important regulatory role in the differential induction of these cytokines and chemokines. This region may be a potential virulence factor of a highly pathogenic PRRSV.

Infection of pigs by virulent and highly virulent PRRSV strains may cause increased mortality, abortion-storms or a severe interstitial pneumonia accompanied by a strong inflammatory response and severe suppurative bronchopneumonia (Rossow, 1998; Blaha, 2000; Rosell et al., 2000). Several viral factors, including the ability to get entry into target cells, viral replication rate, damage to host cells and induction of cell death or specific immune response, may determine the pathogenicity and virulence of a specific strain. Since the re-emergence of virulent strains, numerous studies have been carried out to define the virulence determinant(s). The presence of discontinuous 30 amino acid deletions in nsp2-coding region in both virulent PRRSV-1 and virulent PRRSV-2 strains was reported in many studies and was considered as genetic markers for virulent strains (Li et al., 2007; Zhou et al., 2008; do et al., 2016; Canelli et al., 2017). However, as these mutations were also found in some low virulent strains, their biological significance remains to be determined (Zhou et al., 2014). Deletion of specific nsp2 epitopes may play a role in modulating host immunity and viral infectivity (Chen et al., 2010), which was supported by a more recent study showing the loss of infectivity of a mutant JXwn06 strain (with a mutation in nsp2), due to changes in the cell tropism (Song et al., 2019). Furthermore, ORF1b region, specifically nsp9 and nsp10, contributes to the fatal *in vivo* and *in vitro* virulence of JXwn06 strain, with residues 586 and 592 of nsp9 being highlighted as critical sites regulating the replication of this virulent PRRSV-2 strain (Xu et al., 2018). In this line, other studies recently identified a role of amino acids 519–544 in nsp9 in the pathogenicity and replication efficiency of the virulent HuN4 PRRSV-2 strain (Zhao et al., 2018). These results were largely in agreement with a previous study using a virulent PRRSV infectious clone (FL12) from the virulent NVSL 97–7,895 PRRSV-2 strain, pointing out that multiple genes, including nsp3-8 and ORF5 regions as the main virulence determinants together with nsp1-3, nsp10-12 (ORF1b) and ORF2, are associated with PRRSV virulence (Kwon et al., 2008). This hypothesis would be also supported by the finding of mutations and deletions in ORF5 as possible viral genetic determinants for virulence in some virulent Asian PRRSV-2 strains, such as HuN4 (Ko Ko et al., 2019). Our observations presented in this study add more supportive evidence that ORF5-encoded GP5 functions as a critical virulence determinant.

The most variable region of PRRSV structural proteins is GP5 protein, and the homology varies greatly between strains of different subtypes. For example, the homology between the American and European strains is generally at the range from only 51 to 55% (Key et al., 2001). Neutralizing activity of PRRSV has been shown to correlate with the level of antibodies against GP5, both *in vivo* and *in vitro* (Wissink et al., 2003; Plagemann, 2004). Currently, several sites in GP5 have been demonstrated to be associated with antibody production, including three B cell epitopes, one conserved non-immunoneutralizing epitope and two CD4 + T cell epitopes reported in this protein (de Lima et al., 2006; Vashisht et al., 2008). Neutralization antibody epitopes were also identified in GP4 and M proteins (Díaz et al., 2005). As most PRRSV GP5 protein contains three or more aspartate-linked glycosylation sites (Mardassi et al., 1995), oligosaccharides may also play a key role in the production of infectious PRRSV virus (Ansari et al., 2006). These aspartate glycosylation sites were also found to be adjacent to neutralizing epitopes, suggesting that they may interfere with the binding of antibody to neutralizing epitopes (Jiang et al., 2007). Glycosylation of GP5 inhibits the production of neutralizing antibodies (Ansari et al., 2006). Neutralizing antibodies appeared to be able to completely neutralize homologous viruses of sows and their piglets (Osorio et al., 2002), but unable to neutralize heterogenous virus isolates (Bautista and Molitor, 1999; Kwang et al., 1999; Ostrowski et al., 2002). In these cases, antibody response only provides weak protection, and long-term low levels of antibodies will lead to antibody-dependent enhancement effect, causing greater harm to the animal (Ostrowski et al., 2002). In this study, NNN33-35 residues in GP5 from the highly pathogenic HUB2 strain may create a putative N-linked glycosylation site. However, as overexpression of both wild type and chimeric GP5 proteins carrying these three amino acids did not show migration shift on SDS-PAGE gel, it is unclear if this site would be indeed modified by glycosylation in virus-infected cells.

Innate immunity is the front line in antiviral immune responses and bridges adaptive immunity against viral infections (Wu and Hur, 2015), PRRSV engages several strategies to evade the porcine innate immune responses. A previous study showed that PRRSV infection inhibited IFN-β production primarily by interfering with the MAVS activation in the RIG-I signaling pathway (Luo et al., 2008). PRRSV nsp4, the 3C-like protease (3CLSP), cleaves MAVS at Glu268 and the endoribonuclease activity of nsp11 degrades MAVS and RIG-I mRNA, inhibiting type I IFN signaling (Dong et al., 2015; Sun et al., 2016). PRRSV infection in swine also causes severe interstitial pneumonia (Morgan et al., 2016), indicating that the inflammatory response plays an important role in infection and pathogenesis of PRRSV (van REETH et al., 1999). Previous studies showed that the expressions of IL-1β, IL-8 and TNF-α were significantly elevated in virulent PRRSV-infected swine (Thanawongnuwech et al., 2004). Similarly, HP-PRRSV generates high levels of inflammatory cytokines including IL-1, IL-6 and TNF-α in peripheral blood (Li et al., 2017), indicating that HP-PRRSV may aggravate inflammation and damage tissues and organs. In addition, in pregnant gilts that were challenged on 85 days of gestation and euthanized 21 days post-infection, expression of cytokine genes was significantly upregulated in the thymus and spleen of the fetuses (Alex Pasternak et al., 2020). PRRSV also upregulates cytokine in PAMs and microglia (Qiao et al., 2011; Chen et al., 2014).

PRRSV N and nsp2 proteins have been reported as activators activating NF-kB during infection, whereas nsp1α, 1β, 2, 4 and 11 are known as suppressors (Lee and Kleiboeker, 2005). The contradictory roles of PRRSV proteins in NF-κB regulation further complicates the pathogenesis of this virus. Suppression of NF-κB may lead to the suppression of type I IFN response and activation of NF-κB may result in the production of proinflammatory cytokines. PRRSV infection of PAM cells activates the NLRP3 inflammasomes, inducing IL-1β production dependent on the TLR4/MyD88/NF-κB signaling pathway, and viral RNA can be sensed by cytosolic RNA sensor DDX19A (Bi et al., 2014; Li et al., 2015). PRRSV E protein was able to increase IL-1β release from LPS-primed PAM cells, while nsp11 may inhibit the secretion of IL-1β (Zhang et al., 2013). The endoribonuclease activity of nsp11 is essential for inhibition of IL-1β production (Wang et al., 2015)and NLRP3 inflammasome in microglia (Chen et al., 2018). PRRSV infection also induces IL-10 expression *in vivo* and *in vitro* (Suradhat et al., 2003; Singleton et al., 2018), and this induction depends on the NF-κB activation and p38 MAPK signaling (Hou et al., 2012). By screening PRRSV structural and nonstructural proteins, GP5 was identified as an IL-10 inducer, and its overexpression induced the phosphorylation of p38 (Hou et al., 2012; Song et al., 2013). In this study, we demonstrate that the regulatory effects of PRRSV-rSP and rSP-HUB2 on inflammatory factors are closely related to GP5. Infection of cells with the original HUB2 isolate would lend more supports to this conclusion, but, unfortunately, was constrained by the unavailability of this viral isolate. As the two recombinant viruses share the same SP backbone with differences in the GP5/M region only, the phenotypic differences observed in this study would be attributable to this region.

In summary, we report the construction of infectious clone systems for PRRSV-rSP vaccine strain and a chimeric strain rSP-HUB2. The two viruses are genetically stable, with the chimeric strain exhibiting growth and fitness advantages. Infection of cells with the two recombinant viruses showed differential regulation of the induction of a number of innate immune and proinflammatory genes in infected cells, which was partially attributable to amino acid residue F16 in GP5(HUB2). Further studies would be required to unravel other mechanisms underlying the growth and fitness advantage of this chimeric virus, in addition to its ability to differentially induce the expression of these innate immune and proinflammatory genes. Overall, this study has identified a potential PRRSV virulence determinant and would be instrumental in studying the pathogenicity of PRRSV and in precise modification of virulent strains for developing live attenuated vaccines against PRRSV infection.

## Data availability statement

The datasets presented in this study can be found in online repositories. The names of the repository/repositories and accession number(s) can be found in the article/Supplementary material.

## Ethics statement

Ethical review and approval was not required for the animal study because PAM cells used in this study were a gift from Zhaoqing Da Huanong Biopharmaceutical Co., Ltd., and were prepared from pigs in the Experimental Animal Center of Xinxing Dahua Agricultural, Poultry and Egg Co., Ltd., Yunfu, China, approved number SCXK (Guangdong) 2018-0019.

## Author contributions

MH, RC, and DL contributed to conception and design of the study. YW, GD, and LW performed the experiments. YW and GD organized the database. YW, GD, and DL performed the statistical analysis. YW wrote the manuscript draft and DL did critical revision. All authors contributed to the article and approved the submitted version.

## Funding

This work was partially supported by National Natural Science Foundation of China grants (31972660 and 32170152) and Zhaoqing Xijiang Innovative Team Foundation of China (grant number P20211154-0202).

## Conflict of interest

MH and LW were employed by the company Zhaoqing Institute of Biotechnology Co., Ltd.

The remaining authors declare that the research was conducted in the absence of any commercial or financial relationships that could be construed as a potential conflict of interest.

## Publisher’s note

All claims expressed in this article are solely those of the authors and do not necessarily represent those of their affiliated organizations, or those of the publisher, the editors and the reviewers. Any product that may be evaluated in this article, or claim that may be made by its manufacturer, is not guaranteed or endorsed by the publisher.
