# Supplementary material for: Construction of an infectious cloning system of porcine reproductive and respiratory syndrome virus and identification of glycoprotein 5 as a potential determinant of virulence and pathogenicity
Source: Front Microbiol. 2023 Jul 20;14:1227485. doi: 10.3389/fmicb.2023.1227485 (PMC10397516; doi:10.3389/fmicb.2023.1227485)
Supplement: Supplementary file 1 [file Data_Sheet_1.pdf]

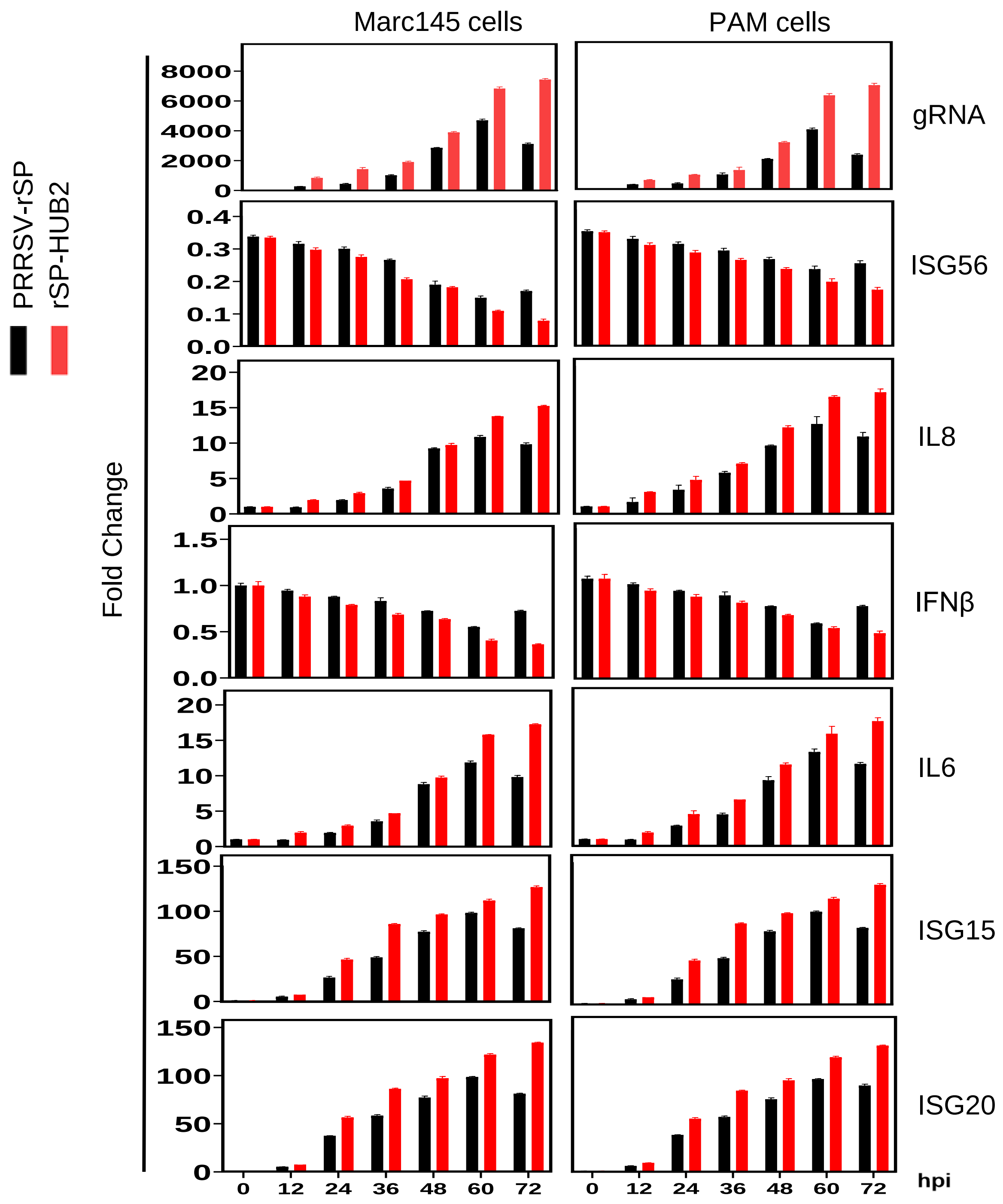

Supplementary figure 1

RT-qPCR analysis of viral gRNA and mRNA levels of IL6, IL8, ISG15, ISG20, IFN-β and ISG56 in Marc-145 and PAM cells infected with PRRSV-rSP and rSP-HUB2, respectively. Cells were infected with PRRSV-rSP and rSP-HUB2, respectively, at an MOI of 1, harvested at indicated time points. Total RNAs were extracted and the levels of viral gRNA and above cytokines/chemokines were determined by RT-qPCR.

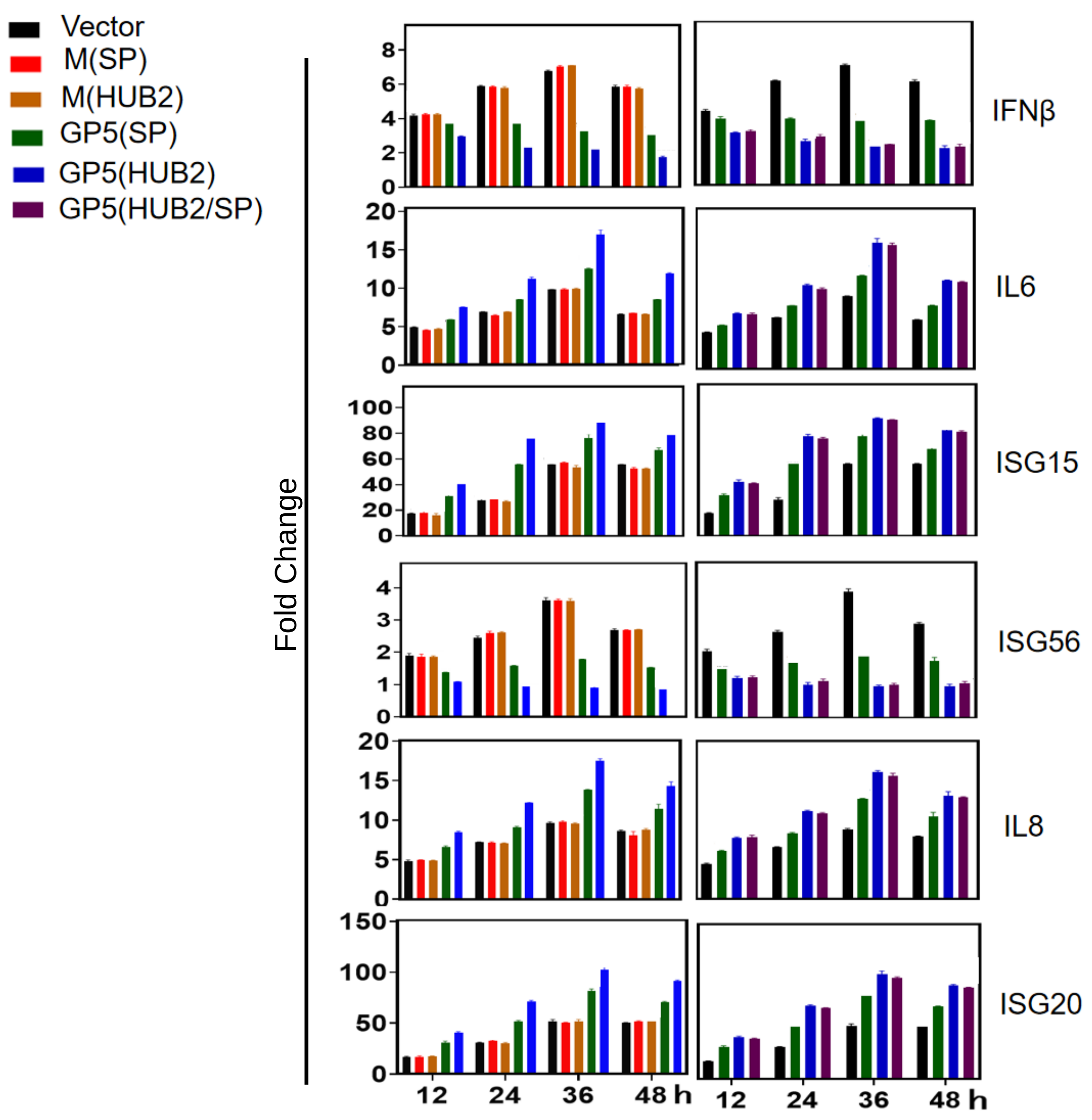

Supplementary figure 2

RT-qPCR analysis of mRNA levels of IL-6, IL-8, ISG15, ISG20, IFN-β and ISG56 in transfected Marc-145 cells. Cells were transfected as described in Figure 4b and harvested at indicated time points post-transfection. Total RNAs were extracted and mRNA levels of above cytokines/chemokines were determined by RT-qPCR.

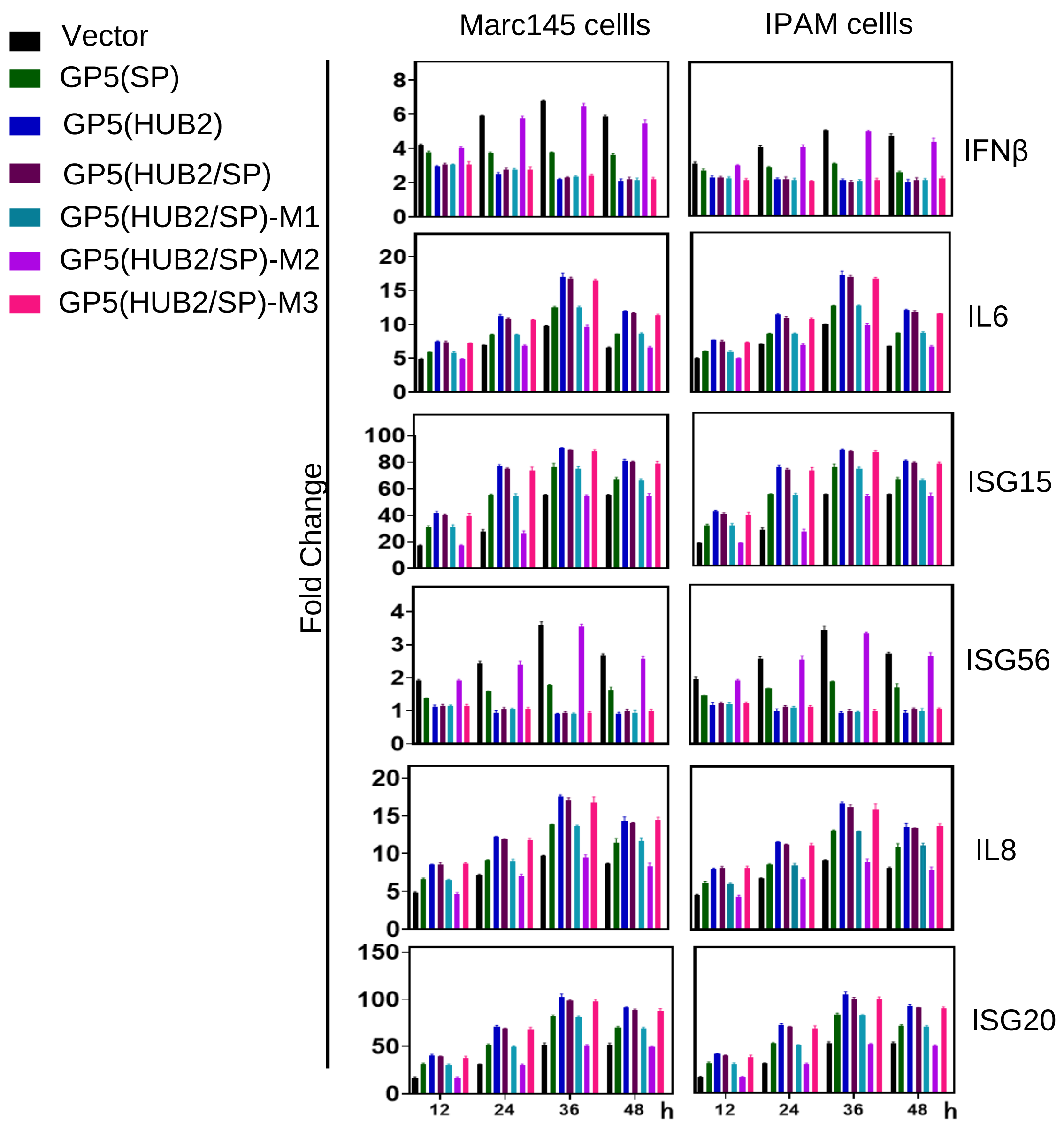

Supplementary figure 3

RT-qPCR analysis of mRNA levels of IL-6, IL-8, ISG15, ISG20, IFN-β and ISG56 in transfected Marc-145 and IPAM cells. Cells were transfected as described in Figure 5b and harvested at indicated time points post-transfection. Total RNAs were extracted and mRNA levels of above cytokines/chemokines were determined by RT-qPCR.
